# Algae *Undaria pinnatifida* Protects Hypothalamic Neurons against Endoplasmic Reticulum Stress through Akt/mTOR Signaling

**DOI:** 10.3390/molecules201219744

**Published:** 2015-11-25

**Authors:** Jongwan Kim, Il Soo Moon, Tae-Won Goo, Seong-Su Moon, Minchul Seo

**Affiliations:** 1 Institute of Medical Research, Dongguk University College of Medicine, Gyeongju 38066, Korea; kimjw3189@naver.com; 2 Department of Anatomy, Dongguk University College of Medicine, Gyeongju 38066, Korea; moonis@dongguk.ac.kr; 3 Department of Biochemistry, Dongguk University College of Medicine, Gyeongju 38066, Korea; gootw@dongguk.ac.kr; 4 Department of Internal Medicine, Dongguk University College of Medicine, Gyeongju 38066, Korea

**Keywords:** *Undaria pinnatifida*, ER stress, apoptosis, autophagy, hypothalamus

## Abstract

Increased endoplasmic reticulum (ER) stress is known to be one of the causes of hypothalamic neuronal damage, as well as a cause of metabolic disorders such as obesity and diabetes. Recent evidence has suggested that *Undaria pinnatifida* (UP), an edible brown algae, has antioxidant activity. However, the neuroprotective effect of UP has yet to be examined. In this study, to investigate the neuroprotective effect of UP on ER stress-induced neuronal damage in mouse hypothalamic neurons, mice immortal hypothalamic neurons (GT1-7) were incubated with extract of UP. ER stress was induced by treating with tunicamycin. Tunicamycin induced apoptotic cell death was compared with the vehicle treatment through excessive ER stress. However UP protected GT1-7 cells from cell death, occurring after treatment with tunicamycin by reducing ER stress. Treatment with UP resulted in reduced increment of ATF6 and CHOP, and recovered the decrease of phosphorylation of Akt/mTOR by tunicamycin and the increment of autophagy. These results show that UP protects GT1-7 cells from ER stress induced cell death through the Akt/mTOR pathway. The current study suggests that UP may have a beneficial effect on cerebral neuronal degeneration in metabolic diseases with elevated ER stress.

## 1. Introduction

The endoplasmic reticulum (ER) provides a unique environment for the synthesis, folding, and maturation of secreted and transmembrane proteins. The ER also plays a critical role toward maintenance of cellular calcium homeostasis. ER stress is an emerging important mechanism induced from imbalance between the protein folding capacity of the ER and the newly synthesized protein load, resulting in accumulation of misfolded protein [1]. To counteract ER stress, the cell activates the unfolded protein response (UPR) to restore cellular homeostasis by clearing the misfolded protein within the ER lumen. Nonetheless, under severe and prolonged ER stress, when homeostasis cannot be restored, UPR activates unique pathways that lead to cell death through apoptosis [2]. Disruption of these physiological functions by ER stress has been implicated in a wide variety of human diseases, including Alzheimer’s disease, Parkinson’s disease, neuronal damage by ischemia, prion disease, cystic fibrosis, obesity, and diabetes mellitus [1,3]. In particular, hypothalamic ER stress has been suggested to cause feeding behavior disorder and glucose dysregulation as for obesity and diabetes [4,5,6,7].

The edible brown algae *Undaria pinnatifida* (UP) is widely distributed in Northeast Asia (China, Japan, and Korea). Fucoidan, a class of fucose-enriched sulfated polysaccharides, derived from UP has been reported to have various biological activities, including anti-tumor, anti-oxidative, anti-inflammatory, anti-viral, and anti-angiogenic properties [8,9]. Recently, UP was shown to attract development and complexity of neurons [10,11]. However, no study to elucidate the direct effect of UP on ER stress and ER stress-induced cell death in hypothalamic neurons has been reported. Therefore, since hypothalamic ER stress had been widely considered as an approach toward prevention of obesity and diabetes, we were interested in whether UP also has a protective effect from ER stress-induced neuronal cell death. In this study, we demonstrated the suppression effect of ethanol extract of UP on ER stress-induced cell death on GT1-7 cells, and attempted to examine the mechanism by which UP has anti-apoptotic activity against ER stress.

## 2. Results

### 2.1. UP Has a Protective Effect in Tunicamycin-Induced Cell Death

**Figure 1:**
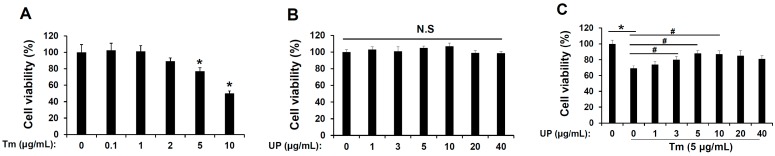
Role of *Undaria pinnatifida* (UP) in endoplasmic reticulum (ER) stress-induced cytotoxicity in the GT1-7 cells. (**A**) GT1-7 cells were treated with tunicamycin (0.1–10 μg/mL) for 24 h, and then cell viabilities were measured with MTT assay. The results are means ± SD (*n* = 3); * *p* values of <0.05 indicate significantly different from treatments without tunicamycin; (**B**) The GT1-7 cells were treated with UP (1–40 μg/mL) for 24 h, and then cell viabilities were measured with MTT assay. The results are means ± SD (*n* = 3); (**C**) The GT1-7 cells were treated with tunicamycin (5 μg/mL) for 24 h with UP (1–40 μg/mL), and then cell viabilities were measured with MTT assay. The results are means ± SDs (*n* = 3); * *p* values of <0.05 indicate significantly different from treatments without tunicamycin. **^#^** *p* values of <0.05 indicate significantly different from treatments with tunicamycin alone.

To evaluate the effect of tunicamycin, ER stress inducer induced cell death in GT1-7 cells, GT1-1 cells were exposed to various concentrations of tunicamycin for 24 h resulted in decreased cell viability in a concentration-dependent manner (Figure 1A). Subsequently, to determine the role of UP in tunicamycin-induced cell death, cells were exposed to various concentrations of UP with tunicamycin. Simultaneous treatment with UP (5–40 μg/mL) and tunicamycin (5 μg/mL) for 24 h resulted in attenuation of tunicamycin-induced cell death (Figure 1B). In addition, no effects on cell viability were observed at the concentrations of UP used in the current study (Figure 1C). These results raised the possibility that UP has a beneficial effect against tunicamycin-induced cell death in GT1-7 cells.

### 2.2. UP Attenuates ER Stress

**Figure 2:**
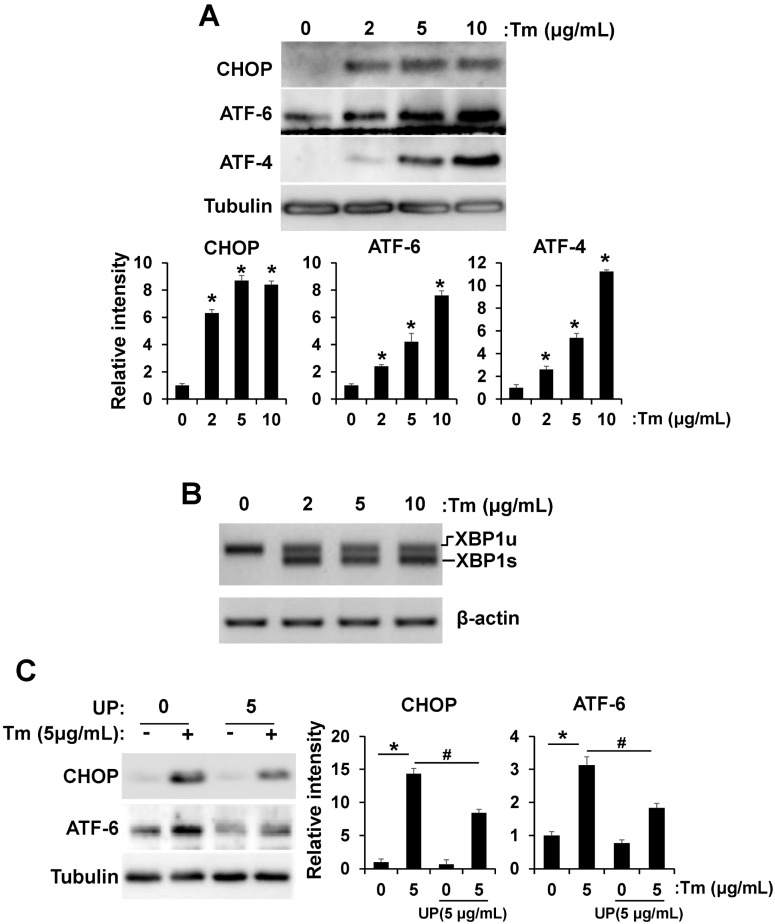
UP attenuates tunicamycin induced ER stress. (**A**) GT1-7 cells were treated with tunicamycin (2–10 μg/mL) and the levels of CHOP, ATF6, and ATF4, ER stress marker, were measured by Western blotting after 24 h. The results are means ± SD (*n* = 3); * *p* values of <0.05 indicate significantly different from without tunicamycin; (**B**) GT1-7 cells were treated with tunicamycin (2–10 μg/mL) and alternative XBP1 mRNA splicing was measured by RT-PCR after 24 h; (**C**) GT1-7 cells were treated with tunicamycin (24 h) with or without UP (5 μg/mL) and the levels of CHOP and ATF6 were measured by Western blotting after 24 h. The results are means ± SD (*n* = 3); * *p* values of <0.05 indicate significantly different from treatments without tunicamycin. ^#^ *p* values of <0.05 indicate significantly different from treatments without UP.

In previous works, tunicamycin increased ER stress markers, such as CHOP, ATF6, ATF4, and XBP-1 splicing in the hypothalamus, often leading to cell death [12,13,14]. Therefore we investigated the expression levels of ER stress-induced CHOP, ATF6, and ATF4 in GT1-7 cells (Figure 2A). Tunicamycin treatment increased levels of CHOP, ATF6, and ATF4 in a concentration-dependent manner up to 2–10 fold. We next determined alternative XBP-1 mRNA splicing, part of the endoplasmic reticulum stress response, using RT-PCR. Exposure to tunicamycin increased alternative XBP-1 mRNA splicing (Figure 2B). Because UP reliably increased the cell viability against tunicamycin induced cell death, we performed additional studies to determine whether UP reduces tunicamycin induced ER stress. As shown in Figure 2C, treatment with UP reduced expression level of tunicamycin induced ER stress marker, specifically CHOP and ATF6, but ATF4 expression was not significantly changed (data not shown).

### 2.3. UP Attenuates ER Stress Induced Apoptotic Cell Death

**Figure 3:**
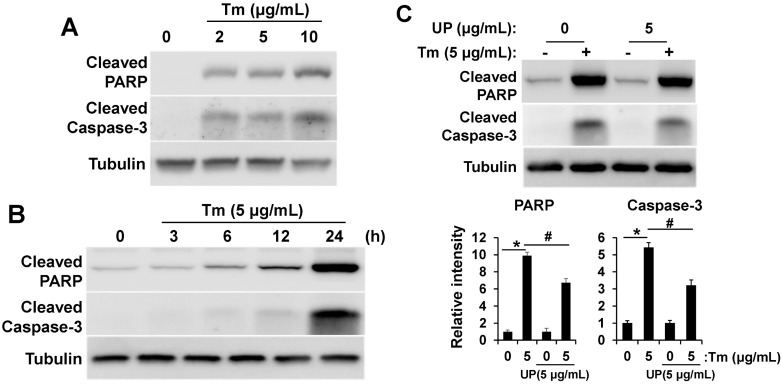
UP alleviates tunicamycin induced apoptotic signaling. (**A**) GT1-7 cells were treated with tunicamycin (2–10 μg/mL) and the levels of cleaved-PARP and cleaved-Caspase-3, apoptosis marker, were measured by Western blotting after 24 h; (**B**) GT1-7 cells were treated with tunicamycin (5 μg/mL) for 3 to 24 h and the levels of cleaved-PARP and cleaved-Caspase-3 were measured by Western blotting; (**C**) GT1-7 cells were treated with tunicamycin (5 μg/mL) with or without UP (5 μg/mL) and the levels of cleaved-PARP and cleaved-Caspase-3 were measured by Western blotting after 24 h. The Tubulin was detected as a loading control. Densitometric analysis results (*lower*) are also shown. The results are means ± SD (*n* = 3); * *p* values of <0.05 indicate significantly different from treatments without tunicamycin. ^#^ *p* values of <0.05 indicate significantly different from treatments without UP.

Interference with ER function leads to accumulation and aggregation of unfolded proteins and initiation of the unfolded protein response (UPR) to restore ER function. However, if the stress is prolonged, apoptotic cell death ensues [14,15]. After determining that UP attenuates tunicamycin-induced ER stress, we hypothesized that UP may regulate apoptotic cell death induced by ER stress. We determined whether tunicamycin induced-apoptosis is modulated by UP treatment using apoptotic markers, such as cleaved-PARP and cleaved-Caspase-3. As shown in Figure 3, the levels of cleaved-PARP and cleaved-Caspase-3 were significantly increased in a concentration (Figure 3A) and time (Figure 3B) dependent manner after treatment with tunicamycin. However these apoptotic signals were decreased with UP treatment (Figure 3C). On the basis of our results, UP seems to alleviate the pro-apoptotic signals through the reduction of ER stress.

### 2.4. UP Increases Cell Viability via Akt/mTOR Signaling

**Figure 4:**
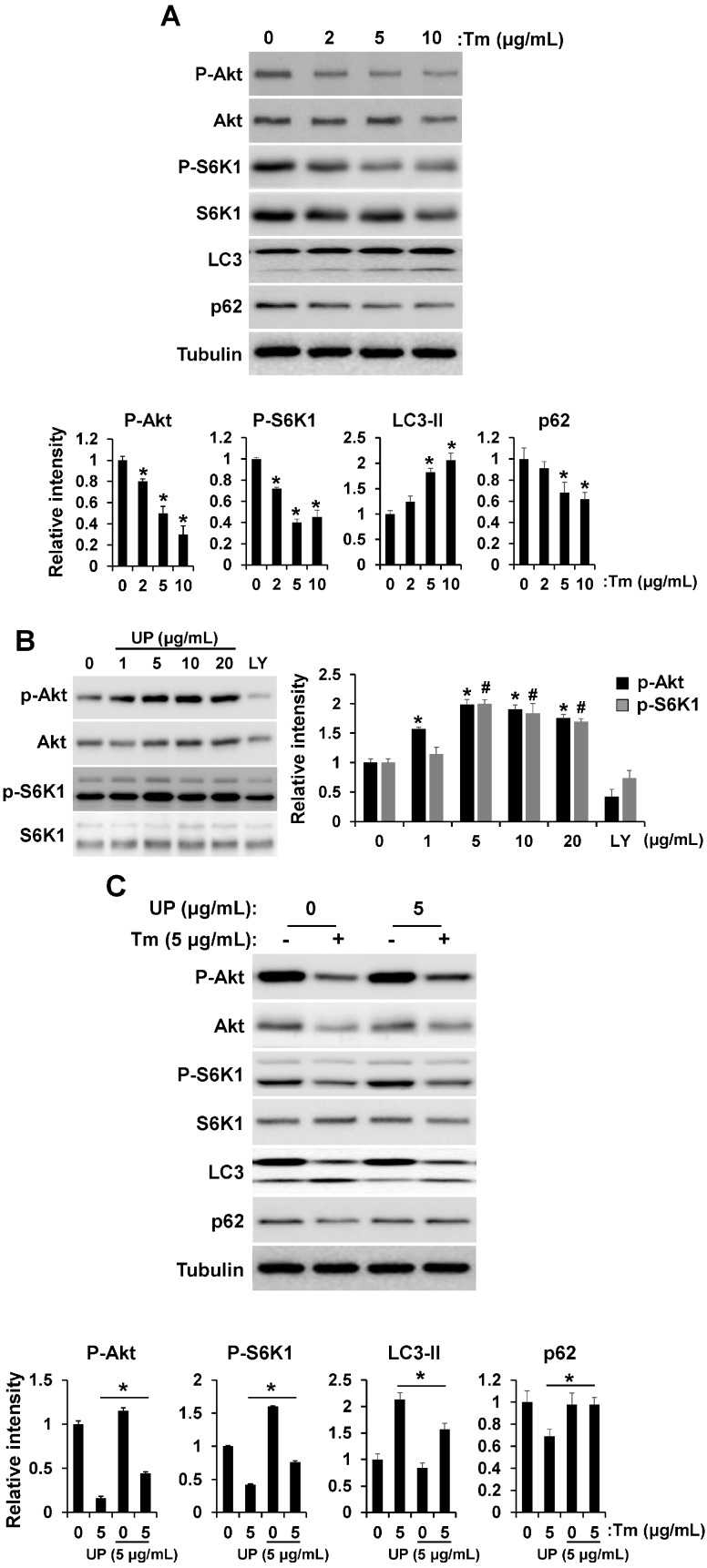
UP increases cell viability through AKT/mTOR signaling. (**A**) GT1-7 cells were treated with tunicamycin (2–10 μg/mL) for 24 h and the levels of phospho-AKT, phospho-p70S6K1, and LC3 were measured by Western blotting. The total AKT, p70S6K1, or Tubulin were detected as a loading control. Densitometric analysis results (*lower*) are also shown. The results are means ± SDs (*n* = 3). * *p* values of <0.05 indicate significantly different from treatments without UP; (**B**) GT1-7 cells were treated with different concentrations of UP for 24 h and the levels of phospho-AKT and phospho-p70S6K1 were measured by Western blotting. The total AKT and p70S6K1 were detected as a loading control. Densitometric analysis results (*right*) are also shown. The results are means ± SDs (*n* = 3). * *p* values of <0.05 indicate significantly different of phospho-AKT from treatments without UP. ^#^ *p* values of <0.05 indicate significantly different of phospho-p70S6K1 from treatments without UP. An amount of 20 μM of LY294002 was used for a positive control; (**C**) GT1-7 cells were incubated with tunicamycin (24 h) with or without UP and the levels of phospho-AKT, phospho-p70S6K1, and LC3 were measured by Western blotting. Densitometric analysis results (*lower*) are also shown. The results are means ± SD (*n* = 3); * p values of <0.05 indicate significantly different from treatment with UP.

**Figure 5:**
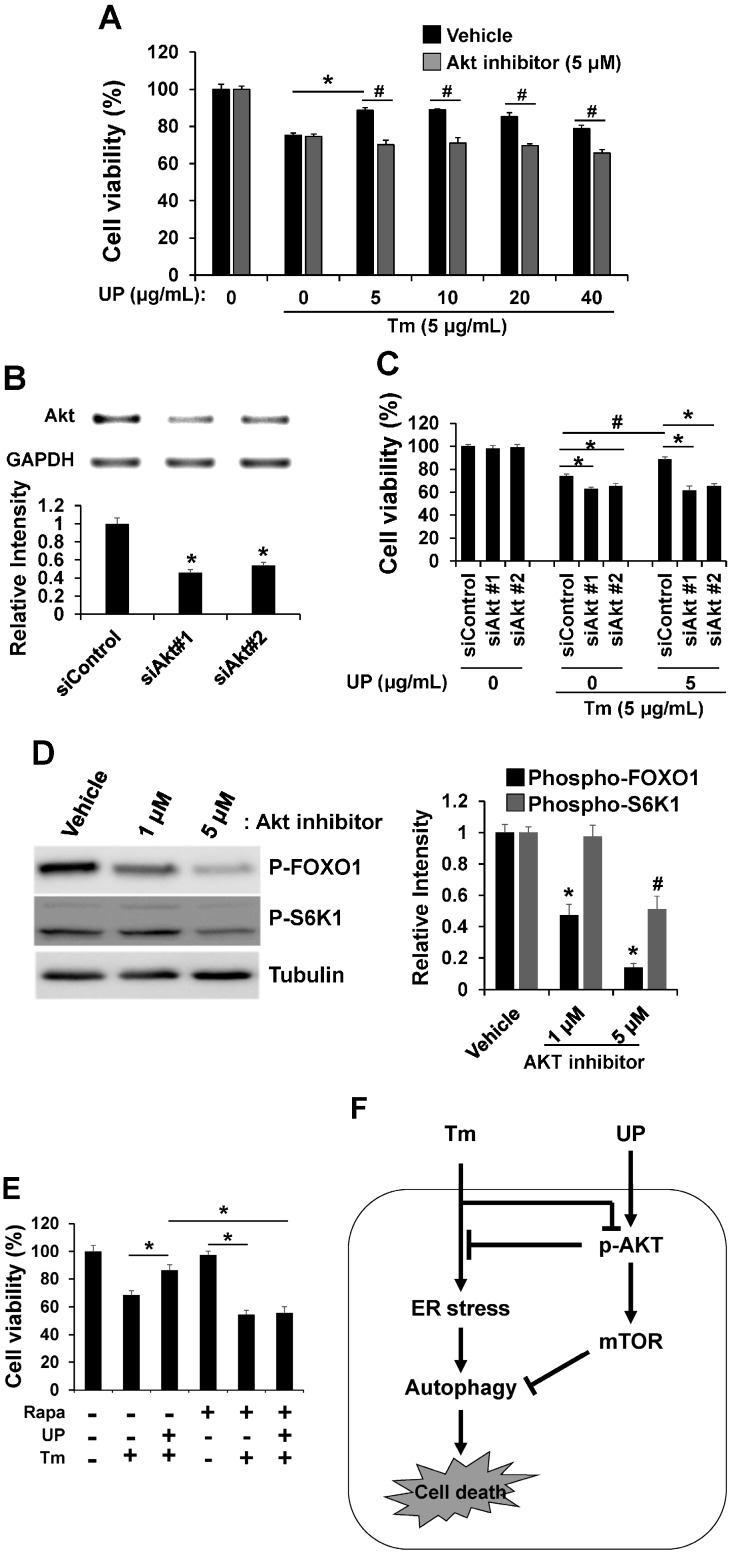
Role of AKT/mTOR signaling in ER stress-induced cytotoxicity. (**A**) GT1-7 cells treated with UP were incubated with tunicamycin (24 h) with or without Akt inhibitor and cell viabilities were then assessed by MTT assay. The results are means ± SDs (*n* = 3); * p values of <0.05 indicate significantly different from treatment with UP. ^#^*p* values of <0.05 indicate significantly different from treatments without inhibitor; (**B**) Knockdown of Akt was demonstrated by RT-PCR analysis. Densitometric analysis results are also shown (*lower*). GAPDH was used as a control. * p values of <0.05 indicate significantly different from control siRNA; (**C**) Expression of Akt in GT1-7 cells was knockdowned using siRNAs against Akt. GT1-7 cells were treated with tunicamycin (5 μg/mL) with or without UP (5 µg/mL) for 24 h, and cell viabilities were measured with MTT assay. The results shown are means ± SDs (*n* = 3). * p values of <0.05 indicate significantly different from control siRNA. ^#^ *p* values of <0.05 indicate significant differences from treatments without UP; (**D**) The levels of phospho-FOXO1 and phospho-p70S6K1 were measured after treatment with Akt inhibitor by Western blotting. Tubulin was detected as a loading control. Densitometric analysis results (*right*) are also shown; (**E**) GT1-7 cells treating UP were incubated with tunicamycin (24 h) with or without rapamycin, mTOR inhibitor, and cell viabilities were assessed by MTT assay. The results are means ± SD (*n* = 3); * *p* values of <0.05 indicate significant differences between the conditions indicated; (**F**) Hypothetical model depicting a central role of UP in ER stress-induced cell death.

Akt/mTOR pathway is well established as a cell survival signal [16]. Crosstalk between the Akt/mTOR and ER stress pathway has emerged [17,18]. Because Akt/mTOR operates both upstream and downstream of ER stress signals, we investigated the effect of tunicamycin on Akt and S6K1 (a marker of mTOR activity) phosphorylation. Tunicamycin treatment decreased phosphorylation of Akt and S6K1 in a concentration dependent manner, which resulted in increase of autophagy (Figure 4A). However, phosphorylation of Akt and S6K1 was increased by UP treatment suggesting that UP can attenuate ER stress-induced apoptotic cell death by increasing Akt/mTOR signaling (Figure 4B). After determining the roles of UP in Akt/S6K1 phosphorylation, we examined whether UP can restore the Akt/S6K1 phosphorylation reduced by tunicamycin. As shown in Figure 4C, UP certainly restored Akt/S6K1 phosphorylation, which resulted in a decrease of autophagy. After determining that UP increases Akt and S6K1 phosphorylation, we hypothesized that UP may regulate cell viability through Akt/mTOR signaling. To determine whether UP can increase the cell viability through Akt/mTOR signaling, we examined cell viability after treatment with the Akt inhibitor and knock-down (Figure 5A–C). As a result, tunicamycin treatment with Akt inhibitor and knock-down strongly suppressed cell viability and the UP effect reducing tunicmycin-induced cell death was abolished with Akt inhibitor and knock-down. Furthermore, in the presence of mTOR inhibitor, rapamycin, drastic decrement of UP induced cell survival was shown under ER stress condition (Figure 5E). These results suggest that Akt/mTOR signaling was necessary for neuroprotective effect of UP against tunicamycin induced cell death.

## 3. Discussion

Hypothalamic ER stress is known to be increased in metabolic diseases such as obesity and diabetes. Pharmacologic or genetic induction of ER stress in the hypothalamus caused central leptin and insulin resistance, resulting in increased food intake, glucose intolerance, and hypertension, whereas reduction of ER stress significantly alleviated these metabolic derangements [19,20]. Previous reports have implicated ER stress-induced cellular dysfunction and cell death are major contributors in many diseases. Therefore making modulators of the ER stress pathway is a potential target for therapeutic strategies [14]. It is well known that prolonged ER stress can lead to cell apoptosis. Several pathways have been identified to explain how cells trigger apoptotic cell death when faced with ER stress. However, the molecular mechanisms underlying ER stress-induced apoptotic cell death in hypothalamic neuronal cells are just emerging.

The findings have shown that Akt, a serine/threonine protein kinase, is a mediator of growth factor-induced cell survival and suppresses apoptotic death [21,22]. Increasing evidence suggests that the PI3K/Akt pathway is involved in ER stress. Activation of Akt in response to ER stress in different cell types has been reported [21]. However, Akt activation was regulated dually on the ER stress according to the ER stress exposure time. Akt was activated on short-term exposure to ER stress but down-regulated on long-term exposure to ER stress [23]. mTOR is essential for Akt-mediated survival signaling [24,25]. That the ER stress can also act upstream of mTORC1 adds a further layer of complexity. Pharmacological induction of UPR leads to rapid activation of the PI3K-Akt-mTORC1 signaling axis [26,27] which depends on the ATF6α branch of the UPR [28]. Prolonged treatment with ER-stress-inducing agents, however, inhibits Akt and mTORC1, which has been attributed to the mTORC1-S6K1-IRS1 negative feedback loop [23,26,27,28,29,30,31]. Suppression of Akt following an extended period of ER stress apparently plays a central role in activation of Ire1α-ASK1-JNK downstream of mTORC1, possibly by derepression of the ASK1 adapt protein TRAF2 [18]. Increasing evidence has shown that the Akt/mTOR pathway is a mediator of growth factor induced cell survival and suppresses apoptotic cell death [21,22,26,27].

On the contrary, in ER stress, downregulation of Akt/mTOR pathway has been reported to be a cytoprotective response to the overload of misfolded or unfolded proteins through autophagy [32]. The previous report supported that PI3K/Akt/mTOR pathway promotes necrotic cell death via suppression of autophagy [33]. Conversely, autophagy induced by ER stress in a normal human colon cell line and in non-transformed murine embryonic fibroblasts contributes to cell death rather than protection. Therefore, the relevance of autophagy in cell survival is not always clear. The impact of autophagy on cell survival during ER stress, whether cytoprotective or proapototic, is likely contingent on the status of cells [34]. Our results reflect its role in promoting cell death. As shown in Figure 1C and Figure 4C, UP can attenuate ER stress-induced cell death through reducing autophagy through upregulation of the Akt/mTOR pathway. These results suggest that autophagy can contribute to ER stress-induced cell death, which may depend on cellular status. However, the exact mechanisms of Akt/mTOR in ER stress-induced cellular dysfunction and cell death are far from fully elucidated [35,36,37].

In summary, our most significant finding is that UP, an ethanol extract of seaweed, shows a neuroprotective effect against ER stress through upregulation of the Akt/mTOR pathway leading to a decrease of autophagy (Figure 5F). Considering the neuroprotective effect of UP on hypothalamic ER stress, our observations suggest that UP may be effective as an anti-obesity and anti-diabetic agent. However, further studies are warranted to clarify which ingredients of UP have those effects as well as to provide strong evidence of the functional roles of UP with Akt/mTOR pathways on hypothalamic ER stress-induced metabolic disease conditions such as obesity and diabetes mellitus.

## 4. Experimental Section

### 4.1. Reagents and Cells

Specific small-molecule inhibitors, such as Akt inhibitor (1L6-hydroxymethyl-chiro-inositol-2-(*R*)-2-*O*-methyl-3-*O*-octadecyl-*sn*-glycerocarbon), were purchased from Calbiochem (La Jolla, CA, USA). Tunicamycin was purchased from Sigma-Aldrich (St Louis, MO, USA) and dissolved in DMSO. All other chemicals, unless otherwise stated, were obtained from Sigma-Aldrich. GT1-7 cells (a generous gift from Dr. Pann-Ghill Suh, UNIST, Korea) were maintained in Dulbecco’s modified Eagle’s media (DMEM) supplemented with 10% heat-inactivated fetal bovine serum (FBS) (Invitrogen, Carlsbad, CA, USA), 100 U/mL penicillin and 100 μg/mL streptomycin (Gibco-BRL, Rockville, MD, USA).

### 4.2. Preparation of *Undaria pinnatifida* Extract

Powdered *Undaria pinnatifida* (UP) was extracted by pouring 95% ethanol into a conical flask containing 5 g of *Undaria pinnatifida* powder at a ratio of 50:1 (*v*/*w*). The mixture was incubated at RT for 24 h under dark conditions. After incubating, the supernatant was filtered and completely dried under a stream of nitrogen gas. The extract was dissolved in dimethyl sulfoxide (DMSO) to make an aliquot (5 mg/mL) and stored at −20 °C for further experiments [10,38].

### 4.3. Cell Viability Test

GT1-7 cells (1 × 10^4^/well in 96-well plates) were cultured, and then cell viability was determined by a 3-(4, 5-dimethylthiazol-2-yl)-2,5-diphenyltetrazolium bromide (MTT) assay. GT1-7 cells were seeded in triplicate at a density of 1 × 10^4^ cells per well on a 96-well plate. At the end of the treatment, the culture media were removed and MTT (0.5 mg/mL) was added, followed by incubation at 37 °C for 2 h in a CO_2_ incubator. After dissolving the insoluble crystals that formed in DMSO, absorbance was measured at 570 nm using a microplate reader (Anthos Labtec Instruments, Wals-Siezenheim, Salzburg, Austria).

### 4.4. Transient Transfection with siRNA

Desalted and pre-annealed siRNA duplexes were purchased from Genolution Pharmaceuticals (Seoul, Korea). The siRNAs were designed using a proprietary algorithm devised by Genolution Pharmaceuticals (Table 1). To knockdown specific gene expression, GT1-7 cells were transfected with siRNAs at a final concentration of 10 nM using LipofectAMINE 2000 (Invitrogen), according to the manufacturer’s instructions. Cells were harvested 24–48 h after transfection, and the potencies of siRNAs to silence gene expression were measured by RT-PCR. A control random sequence siRNA was also purchased from Genolution Pharmaceuticals and used as negative control.

**Table 1:** Sequences of siRNAs.

Target Genes	Symbols	GenBank No.		siRNA Sequences
Control siRNA				Forward, 5’-ACGUGACACGUUCGGAGAAUU-3’ Reverse, 5’-UUCUCCGAACGUGUCACGUUU-3’
Thymoma viral proto-oncogene (Akt)				
Thymoma viral proto-oncogene	*akt*	NM_009652	#1	Forward, 5’-GAACGAUGGCACCUUUAUUUU-3’ Reverse, 5’-AAUAAAGGUGCCAUCGUUCUU-3’
			#2	Forward,5’-GGUUCUUUGCCAACAUCGUUU-3’ Reverse, 5’-ACGAUGUUGGCAAAGAACCUU-3’

### 4.5. Reverse Transcription-PCR

Total RNA was extracted from GT1-7 cells in 6-well plates using TRIzol reagent (Invitrogen), according to the manufacturer’s instructions. Reverse transcription was performed using Superscript II (Invitrogen) and oligo (dT) primer, and the following oligonucleotide primers were used for RT-PCR: *Akt1* forward, 5′-CTGTCTCGAGAGCGTGTGTT-3′; *Akt1* reverse, 5′-AACAGCTTCTCGTGGTCCTG-3′; *Akt2* forward, 5′-ATGACTATGGGCGAGCAGTG-3′; *Akt2* reverse, 5′-TATCGGTCTGGGGGTGTGAT-3′; β*-actin* forward, 5′-AGGGAAATCGTGCGTGACAT-3′; β*-actin* reverse, 5′-CGGACTCATCGTACTCC TGC-3′. PCR amplification was performed at an annealing temperature of 55 °C for 30 cycles. PCR was performed using a DNA Engine Tetrad Peltier Thermal Cycler (MJ Research, Waltham, MA, USA). For analysis of PCR products, 10 μL of each PCR reaction product was electrophoresed on 1% agarose gel followed by ethidium bromide staining and detection under UV light. β-*actin* was used as internal control.

### 4.6. Western Blot Analysis

For Western blot analysis of GT1-7 cells, cells (4 × 10^5^) were seeded on to 6-well culture plates 1 day before incubation with UP extract or tunicamycin. After treatment, cells were lysed in triple-detergent lysis buffer (50 mM Tris-HCl, pH 8.0, 150 mM NaCl, 0.02% sodium azide, 0.1% SDS, 1% NP-40, 0.5% sodium deoxycholate, and 1 mM phenylmethylsulfonyl fluoride). Protein concentrations in cell lysates were determined using the Bio-Rad protein assay kit (Bio-Rad, Hercules, CA, USA). Equal amounts of protein were separated by 10% SDS-PAGE and transferred to PVDF membranes (Amersham Biosciences, Piscataway, NJ, USA). Membranes were blocked with 5% skimmed milk and sequentially incubated with primary antibodies [rabbit polyclonal anti-phospho-Akt (Ser473) antibody (Cell Signaling Technology, Danvers, MA, USA); rabbit polyclonal anti-Akt antibody (Cell Signaling Technology); rabbit polyclonal anti-phospho-FOXO1 (Ser256) antibody (Cell Signaling Technology); rabbit polyclonal anti-FOXO1 antibody (Cell Signaling Technology); rabbit polyclonal anti-phospho-p70S6 kinase (Thr389) antibody (Cell Signaling Technology); rabbit polyclonal anti-p70S6 kinase (Cell signaling Technology); anti-LC3 and monoclonal anti-α-tubulin clone B-5-1-2 (Sigma)], and HRP-conjugated secondary antibodies (anti-rabbit or anti-mouse IgG; Amersham Biosciences), and then detected using an ECL detection kit (Invitrogen).

### 4.7. Statistical Analysis

Unless stated otherwise, results are presented as the mean ± SD of three or more independent experiments. The student’s *t-* test or one-way ANOVA with Dunnett’s multiple-comparison test was used for comparison of treatments. SPSS version 19.0K (SPSS Inc., Chicago, IL, USA) was used for the analysis, and *p* value differences of <0.05 were considered statistically significant.

## 5. Conclusions

UP, an ethanol extract of *Undaria pinnatifida*, shows a neuroprotective effect against ER stress through upregulation of the Akt/mTOR pathway leading to a decrease of autophagy. Our observations suggest that UP may represent a viable therapeutic option as an anti-obesity and anti-diabetic agent.
